# Supplementary material for: Dendritic Cell-Based Vaccines Recruit Neutrophils to the Local Draining Lymph Nodes to Prime Natural Killer Cell Responses
Source: Cells. 2022 Dec 28;12(1):121. doi: 10.3390/cells12010121 (PMC9818417; doi:10.3390/cells12010121)
Supplement: Supplementary file 1 [file cells-12-00121-s001.zip › cells-2107226-supplementary.pptx]

## Slide 1
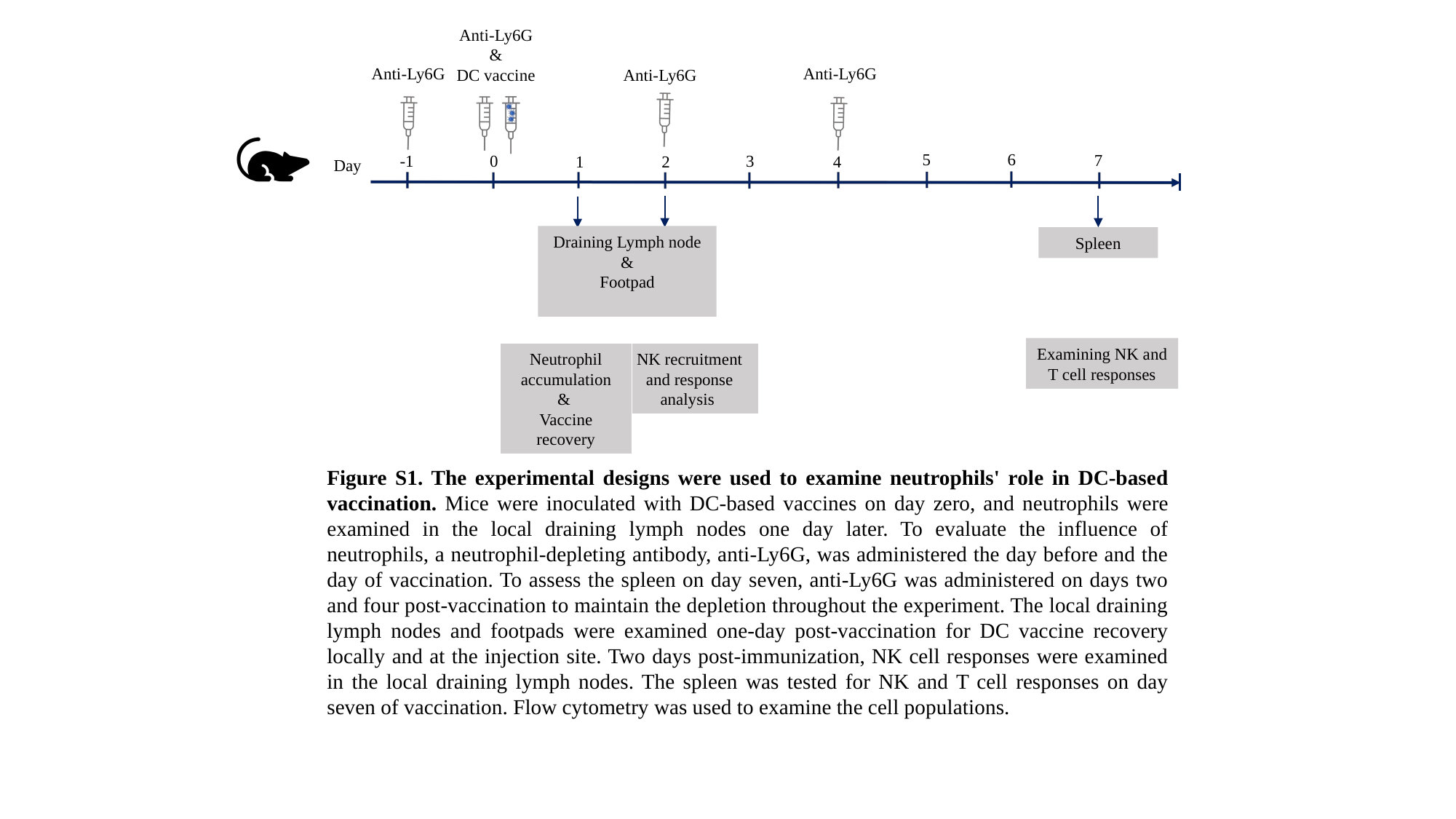

Anti-Ly6G
&
DC vaccine
Anti-Ly6G
Anti-Ly6G
Anti-Ly6G
5
6
7
-1
0
3
1
2
4
Day
Draining Lymph node
&
Footpad
Spleen
Examining NK and T cell responses
Neutrophil accumulation
&
Vaccine recovery
NK recruitment and response analysis
Figure S1. The experimental designs were used to examine neutrophils' role in DC-based vaccination. Mice were inoculated with DC-based vaccines on day zero, and neutrophils were examined in the local draining lymph nodes one day later. To evaluate the influence of neutrophils, a neutrophil-depleting antibody, anti-Ly6G, was administered the day before and the day of vaccination. To assess the spleen on day seven, anti-Ly6G was administered on days two and four post-vaccination to maintain the depletion throughout the experiment. The local draining lymph nodes and footpads were examined one-day post-vaccination for DC vaccine recovery locally and at the injection site. Two days post-immunization, NK cell responses were examined in the local draining lymph nodes. The spleen was tested for NK and T cell responses on day seven of vaccination. Flow cytometry was used to examine the cell populations.

## Slide 2
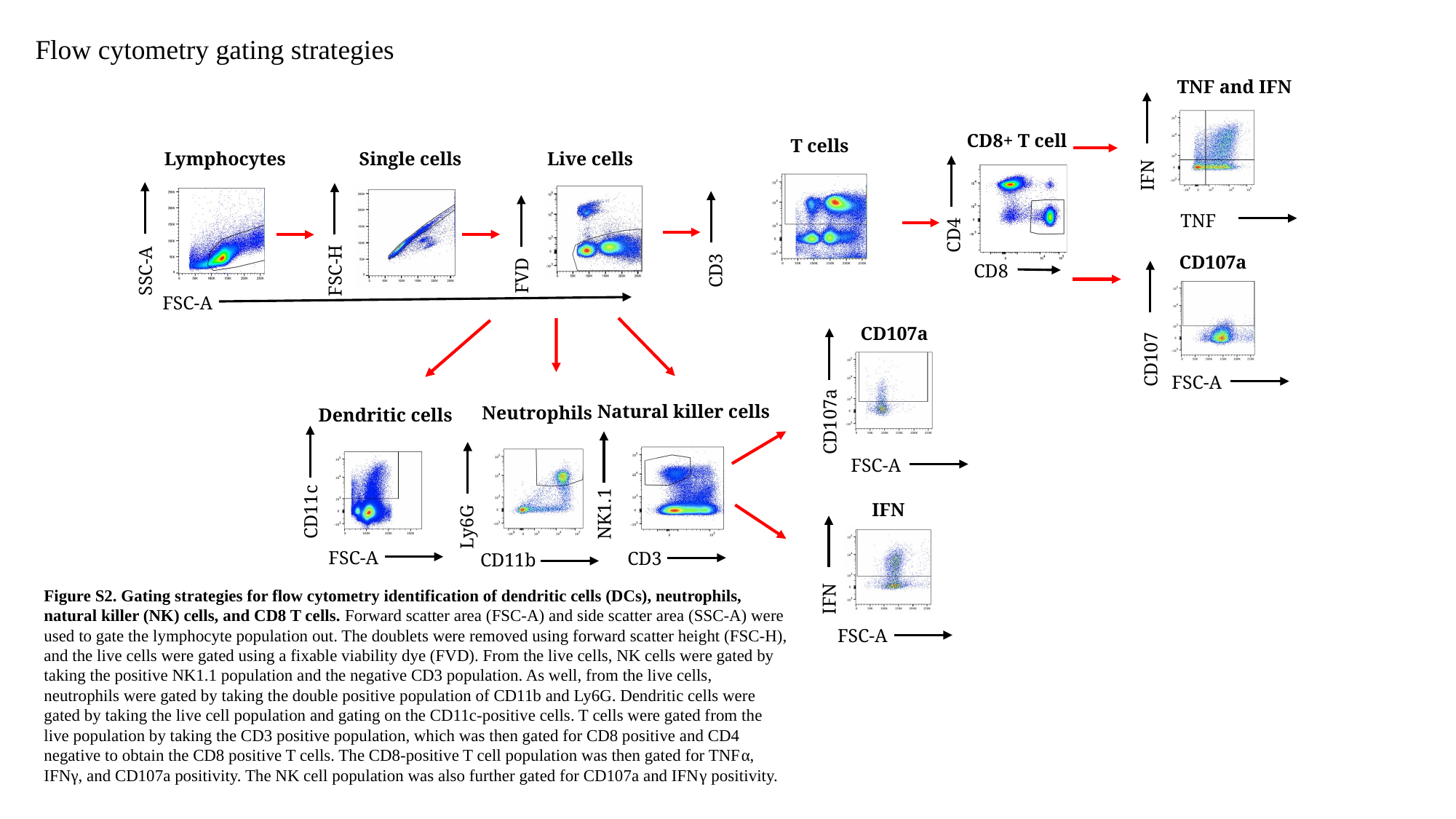

Flow cytometry gating strategies
CD8+ T cell
T cells
Live cells
Lymphocytes
Single cells
CD4
CD3
FVD
CD107a
SSC-A
FSC-H
CD8
FSC-A
CD107a
FSC-A
CD107a
Natural killer cells
Neutrophils
Dendritic cells
FSC-A
CD11c
NK1.1
Ly6G
FSC-A
CD3
CD11b
Figure S2. Gating strategies for flow cytometry identification of dendritic cells (DCs), neutrophils, natural killer (NK) cells, and CD8 T cells. Forward scatter area (FSC-A) and side scatter area (SSC-A) were used to gate the lymphocyte population out. The doublets were removed using forward scatter height (FSC-H), and the live cells were gated using a fixable viability dye (FVD). From the live cells, NK cells were gated by taking the positive NK1.1 population and the negative CD3 population. As well, from the live cells, neutrophils were gated by taking the double positive population of CD11b and Ly6G. Dendritic cells were gated by taking the live cell population and gating on the CD11c-positive cells. T cells were gated from the live population by taking the CD3 positive population, which was then gated for CD8 positive and CD4 negative to obtain the CD8 positive T cells. The CD8-positive T cell population was then gated for TNFα, IFNγ, and CD107a positivity. The NK cell population was also further gated for CD107a and IFNγ positivity.
FSC-A
